# Neurology of cognition and social behavior. A narrative review of neurobiological bases and clinical aspects

**DOI:** 10.1590/1980-5764-DN-2025-0301

**Published:** 2026-02-16

**Authors:** Raimundo Nonato Campos-Sousa, Kelson James Almeida

**Affiliations:** 1 Universidade Federal do Piauí, Departamento de Neurologia, Teresina PI, Brazil.

**Keywords:** Social Cognition, Social Behavior Disorders, Neurobiology, Neuropsychological Tests, Cognição Social, Transtornos do Comportamento Social, Neurobiologia, Testes Neuropsicológicos

## Abstract

Cognitive and behavioral processes of a social nature emerge in childhood and remain efficient throughout life. An increase in the prevalence of neurocognitive and psychiatric disorders has been noted, with consequent difficulty in understanding facial and body expressions, as well as perceiving emotions. The aim of this narrative or non-systematic review was to provide an overview of the neurobiology and neural bases of cognition about social behavior. We reviewed the neural correlates involved in social perception, empathy, theory of mind (ToM) and decision-making. We also discussed the clinical setting, and recommended tests to evaluate social signs, empathy, ToM process, and social decision-making. Neuropsychological assessment and clinical evaluation of social cognition is an increasing field to evaluate human cognition, with interesting findings added to neurologist practice.

## RESUMO.

Processos cognitivos e comportamentais de natureza social apresentam importância crescente desde a infância e permanecem eficientes durante toda a vida. Tem sido observado um aumento da prevalência de transtornos neurocognitivos e patologias psiquiátricas, com consequentes dificuldades de entendimento das expressões corpóreas e faciais, assim como de percepção das emoções. O objetivo desta revisão narrativa ou não sistemática é realizar um estudo de visão geral sobre a neurobiologia e as bases neurais da cognição no que tange ao comportamento social. Revisamos os correlatos neurais envolvidos na percepção social, empatia, teoria da mente e processos de tomada de decisão. Além disso, discutimos o contexto da prática clínica e os testes recomendados para avaliar os sinais sociais, empatia, processos de teoria da mente e de tomada de decisão social. A avaliação neuropsicológica e clínica da cognição social é um campo crescente na avaliação da cognição humana, com achados interessantes adicionados a prática do neurologista.

## INTRODUCTION

Navigating the social world was the evolutionary force that propelled *homo sapiens* to mental supremacy over all living beings. Our attitudes and behaviors are continually shaped by social stimuli. This is followed by appropriate decisions and attitudes to maintain relationships within a society^1^ ^,^ ^2^. These actions and skills enable us to live in a better, more humane and more decent world^2^ ^,^ ^3^. However, the growing aging population, along with unhealthy lifestyle habits and adverse socio-environmental conditions, contributes to a greater prevalence of social cognitive impairments among older adults^4^. These impairments often affect understanding of facial and body expressions, as well as the perception of emotions, humor, sarcasm, and irony^4^. In general, these conditions impair the ability to infer people’s intentions and feelings. This results in the inability to make appropriate decisions that respect social rules, as well as difficulties in forming and maintaining lasting relationships^3^ ^,^ ^4^.

Available information in the fields of neuroscience and neuropsychology allows for a better understanding of the neurology of social behavior. This non-systematic review provides an overview of current knowledge and foundational theories, with the intent of gathering information on the neurobiology and neural bases of cognition and social behavior. The goal is to motivate and facilitate the understanding of social neuropsychology for neurologists and clinicians, as well as to review the domains of social cognition (SC) that should be routinely examined, and to present the main neuropsychological evaluation tools for clinical assessment. As a narrative review was performed, the authors presented interpretations and informed opinions throughout the text, based on available evidence, aiming to integrate knowledge and promote a critical understanding of SC.

## NEUROBIOLOGY OF SOCIAL COGNITION

Cognitive and behavioral processes of a social nature emerge in childhood and remain efficient throughout life^1^, and many of these skills remain stable or even improve with age, benefiting everyday activities^2^ ^,^ ^3^ ^,^ ^4^.

“Loneliness is a beast, loneliness devours”, said Brazilian composer and musician Alceu Valença in 1984. Several studies have shown that social isolation results in greater morbidity and mortality^5^. In a recent review, it was stated that social isolation compromises planning capacity, memory, hormonal homeostasis, as well as resilience to physical and mental illness^6^. Social stimulation favors the formation of new neural networks, microcircuits and synaptic connections that seem to have neuroprotective effects against neurodegenerative pathologies^6^ ^,^ ^7^.

The social environment has transformed our neurobiology. The human brain has become specialized in processing social stimuli through SC, which uses neural, genetic, hormonal and cellular mechanisms in social interaction^8^. These complex mechanisms process perceived social information and structure emotional relevance for understanding the cognitive and emotional states of others^9^. This social brain*,* considering our goals and those of our peers, selects appropriate behaviors and makes decisions according to sociomoral norms in attitudes that are essential for good social coexistence^9^ ^,^ ^10^.

## GENETICS

The interaction between genetics and the environment determines psychological characteristics and social behavior. Studies with twins pointed out that human social behavior is partly under genetic control^11^. The neuropeptides oxytocin and vasopressin, and the neurotransmitters serotonin and dopamine, play important roles in behavior. Oxytocin (OXTR gene) acts in the amygdala to modulate emotions and strengthen prosocial behavior^12^ ^,^ ^13^. The serotonin transporter gene (SLC6A4) regulates mood and emotions, while the dopamine receptor gene (DRD4) acts on empathy, motivation and reward^14^ ^,^ ^15^ ^,^ ^16^.

Approximately half of antisocial behavior is attributed to genetic predispositions, and the rest to environmental effects^17^. The catechol o-methyltransferase (COMT) gene modulates dopamine levels in the prefrontal cortex and some polymorphisms are associated with subtypes of antisocial behavior^16^. Other genes involved in antisocial behavior include the monoamine oxidase A gene (MAO.A), the dopamine active transporter protein gene (DAT.1), and the DRD.2 gene which encodes the D2 dopamine receptor^18^. The 5 HTTLPR, a polymorphism of SLC6A4, a serotonin transporter gene, has been associated with the development of borderline antisocial personality disorder^19^, as well as the FOXP2 gene, expressed in areas involved in social interaction and whose mutations are associated with antisocial behavior^19^ ^,^ ^20^.

## THE NEURAL BASES

Understanding the structural bases and neural networks of SC is a somewhat arduous and still fragmented process because cognitive processing in broad regions of the brain integrates SC^8^. Various structures work together, including the medial prefrontal cortex and dorsolateral prefrontal cortex, the anterior cingulate cortex, the hippocampus, the amygdala, the anterior insula, the inferior frontal gyrus, and the superior temporal sulcus. These structures regulate and process social behavior, emotions and socio-moral decisions^8^. The anterior and ventrolateral temporal cortices, medial and lateral prefrontal cortices, along with the posterior precuneus, are crucial for semantic memories such as learning etiquette, social rules and interpreting social signals^7^.

We reviewed the neural correlates involved in *social perception, empathy, theory of mind (ToM)* and *decision-making*, cognitive skills studied in clinical practice whose characteristics will be described below. The neural bases of *social perception* comprise the extrastriate body area in the lateral occipito-temporal cortex that responds to body images^21^, the fusiform facial area responsible for the perception of facial expressions, and the prefrontal cortex that identifies and assesses the relevance of social stimuli, as well as interpreting gaze and body posture^7^.

The neural bases of empathy, represented by brain regions such as the anterior cingulate cortex, amygdala, insula, prefrontal cortex, and somatosensory areas, comprise a wide diversity of networks involved in empathic processes^21^. Mirror neurons simulate the actions and intentions of others, playing a moderate supporting role in empathy as processed in various brain regions, including the inferior frontal gyrus, inferior parietal lobe, fusiform area, and superior temporal sulcus^22^ ^,^ ^23^.

Brain regions involved in *mentalization* or ToM processes include the medial prefrontal cortex associated with inference of others’ mental states^24^, the temporal-parietal junction related to understanding and attributing mental states, such as beliefs and intentions of others, and the superior temporal sulcus, involved in processing social actions^21^. Among the brain areas involved in *decision-making*, the ventromedial and dorsolateral prefrontal cortices, as well as the anterior cingulate cortex, stand out as structures involved in mentalization processes and morality, which are necessary for making social decisions^25^ ^,^ ^26^.

## NEURAL NETWORKS

**Figure 1.**
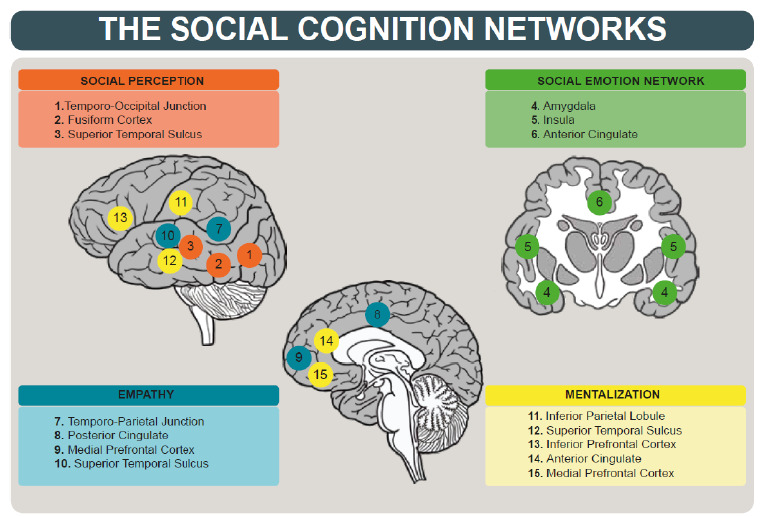
The social cognition networks are presented in accordance with the main social cognition domains.

The previous role of isolated neural structures in SC was questioned, and it the existence of four central neural networks in social processing was proposed: a network activated during the perception of the actions and emotions of others, another network centered around the amygdala and involved with emotional responses to social stimuli, a third that is recruited when individuals have social understanding and empathy, and, finally, a mentalization network that is activated with the inference of the internal states of others^8^. This network view still seems simplistic compared to the findings of new connection maps or connectomes among social integrative networks. Recent reviews have demonstrated that, while certain cortical areas are quite specific to the processing of particular functions, social cognition is processed by intrinsically connected neural networks (ICNs) that interact through complex and flexible connections between various brain structures^27^. Usually, behavioral and cognitive processing utilizes an intricate pattern of interactions (Figure 1) between brain structures^28^_._

A comprehensive review of the specific and critical social cognitive functions of ICNs is highlighted together with their implications in neurodegeneration and neuropathogenesis of dementias^27^. This review suggested that the salience network (SN), connected with the insula, amygdala and cingulum, responds to relevant and salient stimuli and strengthens motivation, attention and decision-making. The SN is primarily involved in the behavioral variant of frontotemporal dementia (BvFTD). The semantic appraisal network (SAN) attributes emotional value to experiences and concepts and is important for understanding and responding to social cues. SAN, which is affected in semantic variant primary progressive aphasia (svPPA) and BvFTD, leads to a series of emotional deficits in patients with dementia^27^.

## CLINICAL ASPECTS

In developmental disorders such as autism spectrum disorder (ASD) and attention deficit hyperactivity disorder, impairment of social skills is frequently observed. In neurodegenerative diseases, such impairments are predominant and early manifested in FTD, particularly in the behavioral variant, and observed in different phases of Alzheimer’s disease, Huntington’s disease, Parkinson’s disease, progressive supranuclear palsy and corticobasal degeneration. Antisocial behavior and sociomoral transgressions can be manifested in these conditions^29^. Social cognition dysfunctions are relevant in psychiatric conditions such as schizophrenia, major depressive disorder and bipolar disorder. The clinical importance of social cognition has been recognized through its inclusion in the Diagnostic and Statistical Manual of Mental Disorders, being considered one of the six fundamental neurocognitive domains^29^ ^,^ ^30^.

Information about social skills should be obtained in the course of the anamnesis from family members, companions and the patient themselves^31^ ^,^ ^32^. In addition to the classic cognitive domains such as attention, memory, visuospatial function and language, the neurologist should assess behavior and social skills. Although social disorders may be difficult to diagnose in the early stages of neurological conditions, structured social cognitive assessment tools can detect subtle alterations, even in presymptomatic stages^31^ ^,^ ^32^. Screening tests and self-report scales are important tools for assessing social cognition disorders in patients with neurodegenerative diseases, neuropsychiatric and psychological disorders^8^. The Brief Social Cognition Battery (BSCB), for example, is an instrument that can be used in primary care settings to assess people with neurocognitive disorders^33^.

## MAIN DOMAINS OF SOCIAL COGNITION

**Figure 2.**
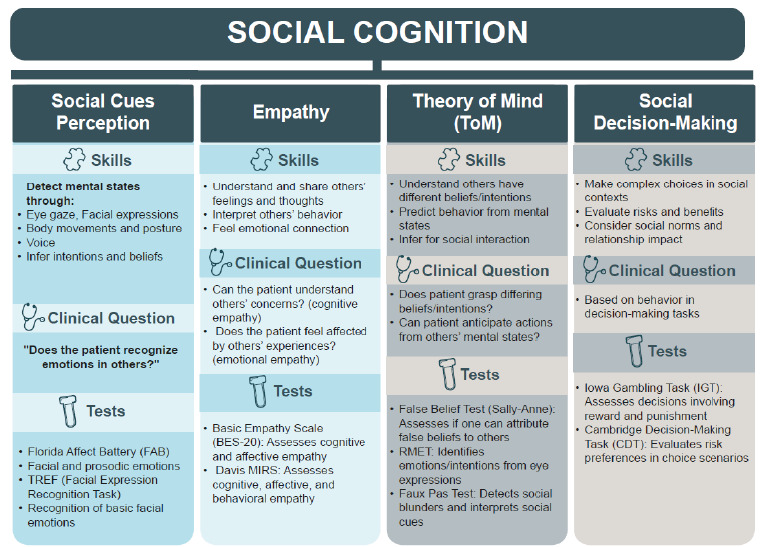
Diagram resuming skills, clinical questions and neuropsychological tools related to the main domains of social cognition.

The clinical assessment of these neuropsychological components is performed through guided questions or structured questionnaires during neurological anamnesis (Figure 2).

### Perception of social signals

Social perception is the ability to capture mental states by observing the social signals emitted through gaze, facial expressions, body movements, posture and voice. Gaze is the most informative social signal^34^. Social perception internalizes others’ affective states (empathy) and enables us to infer and interpret others’ mental states, beliefs and intentions in mentalization processes^35^. During the neurological anamnesis, patients and caregivers should be asked about their ability to recognize basic emotions in other people such as joy, sadness, fear, anger, disgust or even admiration and distraction^36^. Below, validated instruments or those adapted to our reality are suggested.

### Suggested tests

• The Florida Affect Battery (FAB) is a set of tests that assesses facial and prosodic perception. It evaluates the emotions such as happiness, sadness, anger, fear and tone of voice. A study conducted in Brazil adapted the FAB into Portuguese^37^;
• The Facial Expression Recognition Task (TREF) assesses the ability to recognize basic facial emotions and evaluates disgust and contempt^38^. It has been adapted for use in the Brazilian population^38^ ^,^ ^39^;
• The Ekman Faces Test is a classic test that consists in presenting facial expressions through photographs, commonly used in clinical and research settings. The aim of this test is to identify the basic emotions expressed in each photograph^40^.

### Empathy

The ability to perceive and share the feelings and thoughts of others, as well as to understand their behavior and feel a connection with others, is called *empathy* ^*38*^. These skills are impaired in some neurocognitive disorders where antisocial behavior may be observed^41^.

During the neurological anamnesis, questions can be asked about understanding the problems and concerns of others (cognitive empathy) and about feelings and concerns or satisfaction with what happens to others (emotional empathy).

### Suggested tests

• The Basic Empathy Scale (BES-20), validated and adapted to assess empathic abilities, is a 20-item self-report measure designed to measure affective empathy and cognitive empathy^42^;
• The Davis Multidimensional Interpersonal Reactivity Scale (MIRS) is a measure of empathy adapted for Brazil, made up of three subscales with seven items each, that evaluates the affective, cognitive, and behavioral components of empathy^43^.

### Theory of mind

We interpret the behavior of others based on understanding of their minds. ToM or mentalization is the understanding of mental states such as beliefs, intentions and knowledge of oneself and others. This allows us to predict behaviors and make inferences that are important for social interaction. ToM plays an important role in prosocial behavior and morality^29^. This ability can be lost early or during the evolution of some neuropathologies, such as the behavioral variant of frontotemporal dementia, Huntington’s disease, Parkinson’s disease and Alzheimer’s disease^9^.

Questions that should be asked during the anamnesis are: Does the patient understand that others may hold beliefs and intentions that differ from theirs? Can the patient predict the behavior of others based on their mental states?

### Suggested tests

• The “False Belief Test”, also known as the “Sally-Anne test”, measures the ability to attribute false beliefs to others. It is suggested for evaluating ASD in children and adolescents. The test consists of a short story that is told to the patient and involves two children, called Sally and Anne. The patient is then asked to answer questions about the story. The goal is to assess whether the child understands that Sally has a false belief about the location of the object she is searching for^44^;
• The Reading the Mind in the Eyes Test (RMET) assesses the ability to perceive the emotions and intentions of others, from images showing only the eyes. The test consists of 36 photos, and participants must choose one of four response options for each image. The options represent emotions or mental states related to the image. The final score ranges from 0 to 36 points, based on the number of correct answers^45^;
• The *Faux Pas* Test assesses the degree of social adaptation and understanding by interpreting social situations. It is administered by presenting the subject with a series of stories and asking if someone in the story did or said something inappropriate and committed a gaffe or *faux pas*. The stories presented require an understanding of why a character should not have said what they did (*faux pas)* ^*46*^.

### Social decision-making

The ability to make social decisions is a complex cognitive skill that, after allowing an understanding of a situation, evaluates various factors such as risks and benefits, consistency with one’s own values and the communication of decisions. These decisions involve empathy and consideration for the demands and perspectives of others, taking into account socio-moral norms and the impact of decisions on interpersonal relationships^47^.

### Some of these tests include

• The Iowa Gambling Task (IGT) is a neuropsychological test that assesses decision-making and has been adapted to Brazilian Portuguese^48^. Test participants are instructed to choose cards from four decks on a screen, each with a different pattern of rewards and punishments. Participants who are able to learn the rules of the game and choose the decks with positive rewards and few punishments obtain a positive end result;
• Cambridge Decision Task. Validated for the Brazilian population^49^, the CDT consists of a series of scenarios in which participants are asked to choose between two options, each with a different level of risk and reward. The assessment focuses on how often the participant chooses the riskier option, providing insights into risk preferences and how these affect decision-making^49^.

In conclusion, social cognition is the set of mental processes that regulate behavior through the ability to perceive, understand and respond to information and social stimuli in order to infer, interact and build social bonds. Social cognitive deficits impair the ability to form and maintain interpersonal relationships, thus eliminating the benefits that social interactions bring to patients with neuropsychological impairments.

Influenced by genetic and environmental factors, social cognition is processed in various brain structures connected through complex neural networks and involves a range of skills, including social perception, empathy, theory of mind and social decision-making. These functions can be examined through self-report screening tests and formal tests.

Social cognition and behavior disorders can be caused by a variety of factors, including developmental disorders, neurodegenerative diseases, and psychiatric conditions. The assessment of social cognitive deficits is just as important as traditional cognitive assessment. Behavioral symptoms start at very early ages, so even in primary care or specialized outpatient clinics, clinicians and neurologists must be attentive and motivated to assess and use existing knowledge to diagnose social disorders and guide treatment in a timely manner.

This review outlined the main tools based on neuropsychological assessment used for the clinical evaluation of social cognition. These tools can be employed to differentiate pathological behavior from normal behavior, contributing to the diagnosis and monitoring of changes in social cognition. In clinical settings, they may assist in differential diagnosis, neuropsychological intervention planning, and rehabilitation. In addition to offering new therapeutic possibilities, they support the reassessment of treatment response, facilitating the monitoring of progress in neurocognitive disorders and dysfunctions. This enables the tracking of changes in social skills and the adjustment of therapeutic strategies according to the patient’s needs.

## DATA AVAILABILITY STATEMENT

The datasets generated and/or analyzed during the current study are available from the corresponding author upon reasonable request.
